# Poor Oral Health and Diet in Relation to Weight Loss, Stable Underweight, and Obesity in Community-Dwelling Older Adults: A Cross-Sectional Study From the JAGES 2010 Project

**DOI:** 10.2188/jea.JE20150144

**Published:** 2016-06-05

**Authors:** Mieko Nakamura, Toshiyuki Ojima, Miyo Nakade, Rika Ohtsuka, Tatsuo Yamamoto, Kayo Suzuki, Katsunori Kondo

**Affiliations:** 1 Department of Community Health and Preventive Medicine, Hamamatsu University School of Medicine, Hamamatsu, Shizuoka, Japan; 1 浜松医科大学健康社会医学講座; 2 Department of Registered Dietitians, Faculty of Human Wellness, Tokaigakuen University, Nagoya, Japan; 2 東海学園大学健康栄養学部管理栄養学科; 3 Department of Home Care Coordinators, National Center for Geriatrics and Gerontology, Obu, Aichi, Japan; 3 国立研究開発法人国立長寿医療研究センター在宅連携医療部; 4 Department of Dental Sociology, Graduate School of Dentistry, Kanagawa Dental University, Yokosuka, Kanagawa, Japan; 4 神奈川歯科大学大学院歯学研究科社会歯科学講座; 5 Department of Policy Studies, Aichi Gakuin University, Nissin, Aichi, Japan; 5 愛知学院大学総合政策学部; 6 Center for Preventive Medical Sciences, Chiba University, Chiba, Japan; 6 千葉大学予防医学センター

**Keywords:** thinness, aged, fewer teeth, food, fruit and vegetables, 痩せ, 高齢者, 少ない歯数, 食品, 果物と野菜

## Abstract

**Background:**

Involuntary weight loss and underweight increase the risks of mortality and disability in older people. However, the association and interaction of poor oral health and dietary intake with body mass index (BMI) have not been elucidated.

**Methods:**

Data were analyzed for 96 794 respondents aged >65 years who were randomly selected from 31 Japanese municipalities in the Japan Gerontological Evaluation Study. Weight loss was defined as ≥2–3 kg of loss over the preceding 6 months. BMI was evaluated in respondents without weight loss. Multiple logistic regression analysis was performed with weight loss, underweight, and obesity as dependent variables and having fewer teeth (<20) and infrequent food intake as independent variables, with adjustment for potential confounders.

**Results:**

Weight loss was associated with having fewer teeth (men: odds ratio [OR] 1.3; 95% confidence interval [CI], 1.2–1.3; women: OR 1.2; 95% CI, 1.1–1.3) and infrequent fruit/vegetable intake (men: OR 1.1; 95% CI, 1.1–1.2; women: OR 1.4; 95% CI, 1.3–1.5) and fish/meat intake (OR 1.2; 95% CI, 1.1–1.3 for both sexes). No interaction was observed between having fewer teeth and food intake. Obesity was associated with the same factors: having fewer teeth (ORs 1.2 and 1.3 for men and women, respectively) and infrequent intake of fruit/vegetables (ORs 1.1 and 1.2 for men and women, respectively) and fish/meat (OR 1.1 for both sexes). Infrequent fruit/vegetable intake showed a higher OR for underweight in women with fewer teeth than for others.

**Conclusions:**

Having fewer teeth and infrequent food intake were associated with both weight loss and obesity. A significant interaction was observed in the associations of having fewer teeth and infrequent food intake with underweight in women.

## Abstract (ja)

**背景：:**

意図しない体重減少や痩せは高齢者の死亡や障害のリスクを増加させる。しかし歯の不健康・食事とボディマスインデックス（BMI）との関連や交互作用は解明されていない。

**方法：:**

日本老年学的評価研究において日本の31市町から無作為に抽出された65歳以上の回答者96,794人のデータを分析した。体重減少は調査時点に先立つ半年間における2-3kg以上の減少と定義した。BMIは体重減少がない者について評価した。体重減少、痩せ、肥満を従属変数、少ない歯数（20本未満）、食品の低頻度摂取を独立変数として、交絡因子を調整した多重ロジスティック回帰分析を実施した。

**結果：:**

体重減少は少ない歯数（男性オッズ比（OR）1.3、95％信頼区間（CI）1.2-1.3、女性OR 1.2, 95% CI 1.1-1.3）、果物・野菜の低頻度摂取(男性OR 1.1、 95% CI 1.1-1.2、 女性OR 1.4、95% CI1.3-1.5)、魚・肉の低頻度摂取(男女OR 1.2, 95% CI 1.1-1.3)と関連していた。少ない歯数と食品摂取の間の交互作用は見られなかった。肥満も同様の要因と関連していた。すなわち少ない歯数(男性OR 1.2、女性OR 1.3)、果物・野菜の低頻度摂取(男性OR 1.1、女性OR 1.2)、魚・肉の低頻度摂取性（男女OR 1.1)であった。歯数が少ない者はそうでない者に比べ、果物・野菜の低頻度摂取は女性の痩せに対して高いオッズ比を示した。

**結論：:**

少ない歯数と食品の低頻度摂取は体重減少と肥満の両方に関連し、女性の痩せとの関連では有意な交互作用が観察された。

## INTRODUCTION

In older people, involuntary (unintentional) weight loss and underweight increase their risks of mortality and mobility disability.^1^^,^^2^ In addition to exhaustion, weakness, slow walking speed, and low physical activity, involuntary weight loss is thought to be one component of “frailty” and increases the risk for adverse health outcomes.^3^

Cross-sectional and cohort studies have found poor oral health to be associated with weight loss,^4^^,^^5^ frailty,^6^^,^^7^ disability^8^^,^^9^ and mortality^8^^,^^10^ in older people. Although the mechanisms underlying these relationships are not well established, systemic inflammation caused by periodontal disease may function as a mediator.^10^ Changes in dietary intake may also mediate the association of poor oral health with weight loss and frailty. Poor oral health due, for example, to fewer remaining teeth or denture use is associated with eating difficulties that restrict food choice,^11^^–^^14^ which in turn can lead to poor nutritional status.^15^^–^^18^

Several studies have found that poor oral health is also associated with underweight^19^^–^^21^ and obesity.^20^^–^^25^ However, only one of these studies has investigated its relationship with dietary intake,^24^ and, to the best of our knowledge, none have investigated the interaction between poor oral health and dietary intake in relation to body mass index (BMI). Therefore, in this study, we aimed to clarify the association and interaction of poor oral health and dietary intake in relation to recent weight loss, underweight, and obesity, while adjusting for physical activity and other potential confounding factors, in a population-based study of community-dwelling older people.

## METHODS

### Study sample

This study analyzed cross-sectional data obtained by the Japan Gerontological Evaluation Study (JAGES) 2010 project,^26^ a mail survey conducted in 31 municipalities across 12 prefectures in Japan between August 2010 and January 2012. The questionnaire was mailed to 169 215 randomly selected individuals who were aged ≥65 years, living independently in the community, and not eligible for benefits from public long-term care insurance. A total of 112 123 respondents replied (response rate: 66.3%), and data for 102 869 respondents with valid information on age, sex, and municipality of residence (valid response rate: 60.8%) were entered into the JAGES database (JAGES2010v3), from which the data analyzed in the present study was obtained.

### Measurements

All data were collected using a self-administered questionnaire. In the study, weight loss was defined as ≥2–3 kg of weight loss in the preceding 6 months and was indicated by an affirmative answer to the questionnaire item, “Has your weight declined by 2–3 kg or more in the past 6 months?” This item was taken from the Kihon Checklist,^27^^,^^28^ a frailty risk assessment developed by the Japanese Ministry of Health, Labour and Welfare and widely used in Japan. The Kihon Checklist is a validated 25-item self-administered questionnaire for identifying frail individuals; the instrument has seven categories, and total score ranges from 0 (no frailty) to 25 (high frailty). The questionnaire item on weight loss was reported to predict the risk of incident long-term care insurance certification (age- and sex-adjusted odds ratio [OR] 1.8; 95% confidence interval [CI], 1.5–2.2).^27^

BMI was calculated as weight (kg) divided by the square of height (m^2^) from the respondents’ self-reported values when these values were within 4 standard deviations (SDs) for age and sex reported in the National Health and Nutrition Survey in Japan.^29^ BMI ≥25.0 was classified as obese, and BMI <18.5 was categorized as underweight, according to the obesity criteria for Japanese people reported by the Japan Society for the Study of Obesity.^30^ Food intake was assessed from frequency of intake of fish/meat and fruit/vegetables per week in the preceding month and categorized as eating <1 time or ≥1 times per day (reference value). As a proxy of oral health, number of remaining teeth was classified as <20 or ≥20 teeth (reference). In regard to potential confounders, denture use was assessed using a yes/no question. Physical activity was estimated by total walking time per day (<30 min or ≥30 min), and smoking status was divided into present smoker or not. Socioeconomic status was assessed by educational attainment (<10 years or ≥10 years) and equivalent annual income. Equivalent annual income was calculated as household yearly income divided by the square root of the household size and was classified as <2 million yen, ≥2 and <4 million yen, ≥4 million yen, or no answer. Respondents with missing data for variables other than equivalent annual income were excluded from the analysis.

### Statistical analysis

The proportion of respondents who experienced weight loss and the proportion of respondents who had not experienced weight loss but were underweight or obese were calculated. Multiple logistic regression analysis was performed, testing weight loss against no weight loss, underweight against normal weight, and obesity against normal weight as dependent variables, with having fewer teeth and food intake as independent variables. That is, those who were underweight were excluded from the analysis of obesity, and those who were obese were excluded from the analysis of underweight. Age-adjusted ORs for each variable and 95% CIs were obtained in model 1. Adjustments were made for present BMI (only in the analysis for weight loss), denture use, walking time, smoking status, educational attainment, and equivalent annual income in model 2 as the main model in this study. All variables shown in Table 1 (ie, number of teeth, fruit/vegetable intake, and fish/meat intake) were additionally included in model 3 for sensitivity analysis. Next, the proportion of respondents in each food intake frequency category was evaluated by sex, number of teeth, and weight status (ie, weight loss; no weight loss; no weight loss, underweight; no weight loss, normal weight; and no weight loss, obese). Multivariate-adjusted ORs for food intake (model 2) were then obtained, with stratification by presence of ≥20 teeth or not. The interaction effect between having fewer teeth and food intake was also evaluated by adding an interaction term in all models. All statistical analyses were performed using IBM SPSS Statistics version 23 (IBM, New York, USA). All tests of significance were two-tailed, with *P* < 0.05 considered significant.

**Table 1.** Proportion of older respondents self-reporting weight loss in the preceding 6 months, underweight and obesity

		*n*^a^	Weight loss (%)	*n*^b^	No weight loss	

					Underweight (%)	Obesity (%)
**Men**						
Total		44 433	15.5	36 431	4.4	23.2
Age, years	65–74	26 042	13.7	22 045	3.2	25.2
	75–84	15 818	17.3	12 532	5.6	21.2
	≥85	2573	22.1	1854	11.0	12.6
Fruit/vegetable intake	Daily	32 649	14.7	27 081	4.3	22.6
	Not daily	10 994	17.7	8735	4.8	25.1
Fish/meat intake	Daily	16 314	13.8	13 646	4.3	22.3
	Not daily	27 050	16.5	21 929	4.5	23.8
Number of teeth	≥20	14 888	12.2	12 839	3.0	22.7
	<20	28 779	17.0	23 038	5.2	23.5
**Women**						
Total		52 361	15.0	42 301	7.7	22.1
Age, years	65–74	29 438	12.3	25 097	6.5	22.3
	75–84	19 039	17.7	14 627	8.4	22.7
	≥85	3884	22.7	2577	15.4	16.6
Fruit/vegetable intake	Daily	42 810	14.1	35 135	7.7	21.2
	Not daily	8375	19.7	6289	7.7	27.4
Fish/meat intake	Daily	21 678	13.3	18 052	8.2	20.0
	Not daily	29 133	16.2	23 118	7.2	23.7
Number of teeth	≥20	16 424	11.4	14 180	7.5	18.8
	<20	34 373	16.5	27 026	7.7	23.7

^a^People with valid answers for weight loss and each variable.

^b^People who had not experienced recent weight loss and had valid answers for height, body weight and each variable.

### Ethical considerations

The JAGES protocol was approved by the Nihon Fukushi University Ethics Committee.

## RESULTS

### Proportion of respondents reporting weight loss, underweight and obesity

Table 1 shows the proportion of respondents reporting weight loss among the 44 433 men and 52 361 women with valid self-reports of weight loss and the proportion of respondents reporting underweight and obesity among the 36 431 men and 42 301 women not reporting weight loss and with valid self-reports of height and body weight. Overall, 15.5% of men and 15.0% of women reported weight loss during the preceding 6 months, with higher proportions found in the oldest group (>85 years), respondents with an infrequent intake of fruit/vegetables and fish/meat, and respondents with fewer teeth (<20). Among the respondents not reporting weight loss, 4.4% of men and 7.7% of women were classified as underweight, and 23.2% of men and 22.1% of women were classified as obese. The proportion of underweight respondents was highest in the oldest group for both sexes, and the proportion of obese respondents was higher in younger group, respondents with an infrequent intake of both food categories, and respondents with fewer teeth.

### Number of teeth and food intake frequency in relation to weight loss, underweight, and obesity

Table 2 shows the ORs for weight loss, underweight, and obesity. Significant associations with weight loss were found for having fewer teeth (men: model 2 OR 1.25; 95% CI, 1.17–1.35; women: model 2 OR 1.24; 95% CI, 1.15–1.34) and infrequent intake of fruit/vegetables (men: model 2 OR 1.15; 95% CI, 1.07–1.22; women: model 2 OR 1.37; 95% CI, 1.26–1.48) and fish/meat (men: model 2 OR 1.18; 95% CI, 1.11–1.26; women: model 2 OR 1.22; 95% CI, 1.15–1.30). These associations were observed in both sexes and in all models.

**Table 2.** Odds ratios for weight loss, underweight, and obesity according to number of teeth and frequency of food intake

		Model 1^a^			Model 2^b^			Model 3^c^		
		
		OR	95% CI		OR	95% CI		OR	95% CI	
**Weight loss, men**										
Number of teeth	<20	1.38	1.30	1.46	1.25	1.17	1.35	1.23	1.14	1.32
Fruit/vegetable intake	Not daily	1.28	1.21	1.36	1.15	1.07	1.22	1.08	1.004	1.16
Fish/meat intake	Not daily	1.25	1.18	1.32	1.18	1.11	1.26	1.14	1.07	1.22
**Weight loss, women**										
Number of teeth	<20	1.38	1.30	1.46	1.24	1.15	1.34	1.21	1.12	1.31
Fruit/vegetable intake	Not daily	1.49	1.41	1.59	1.37	1.26	1.48	1.29	1.19	1.40
Fish/meat intake	Not daily	1.26	1.19	1.32	1.22	1.15	1.30	1.14	1.07	1.22
**Underweight, men**										
Number of teeth	<20	1.56	1.38	1.76	1.47	1.27	1.69	1.44	1.25	1.66
Fruit/vegetable intake	Not daily	1.23	1.09	1.38	1.14	0.999	1.29	1.12	0.97	1.28
Fish/meat intake	Not daily	1.08	0.97	1.20	1.02	0.91	1.15	0.97	0.86	1.09
**Underweight, women**										
Number of teeth	<20	0.96	0.89	1.04	0.96	0.86	1.06	0.96	0.87	1.06
Fruit/vegetable intake	Not daily	1.07	0.97	1.19	1.05	0.93	1.19	1.15	1.01	1.31
Fish/meat intake	Not daily	0.90	0.84	0.97	0.91	0.84	0.99	0.90	0.82	0.98
**Obesity, men**										
Number of teeth	<20	1.15	1.09	1.22	1.15	1.08	1.22	1.15	1.08	1.22
Fruit/vegetable intake	Not daily	1.14	1.08	1.21	1.07	1.01	1.14	1.05	0.99	1.13
Fish/meat intake	Not daily	1.09	1.03	1.14	1.06	1.004	1.12	1.04	0.98	1.10
**Obesity, women**										
Number of teeth	<20	1.39	1.32	1.46	1.34	1.25	1.43	1.32	1.24	1.42
Fruit/vegetable intake	Not daily	1.42	1.34	1.51	1.25	1.16	1.35	1.20	1.11	1.30
Fish/meat intake	Not daily	1.23	1.17	1.29	1.11	1.05	1.18	1.06	0.998	1.13

CI, confidence interval; OR, odds ratio.

^a^Adjusted for age.

^b^Further adjusted for walking time, smoking, educational attainment, equivalent annual income, denture use, and body mass index (only in the analysis for weight loss).

^c^Further adjusted for number of teeth, fruit/vegetable intake, and fish/meat intake.

Having fewer teeth was significantly associated with underweight in men in all models (model 2 OR 1.47; 95% CI, 1.27–1.69). Infrequent intake of fruit/vegetable was also marginally associated with underweight in men (model 2 OR 1.14; 95% CI, 1.00–1.29), but infrequent intake of fish/meat was not (model 2 OR 1.02; 95% CI, 0.91–1.15). Conversely, having fewer teeth and infrequent fruit/vegetable intake were not associated with underweight in women, while infrequent fish/meat intake was inversely associated with underweight (model 2 OR 0.91; 95% CI, 0.84–0.99).

Having fewer teeth (men: model 2 OR 1.15; 95% CI, 1.08–1.22; women: model 2 OR 1.34; 95% CI, 1.25–1.43) and infrequent intake of fruit/vegetables (men: model 2 OR 1.07; 95% CI, 1.01–1.14; women: model 2 OR 1.25; 95% CI, 1.16–1.35) and fish/meat (men: model 2 OR 1.06; 95% CI, 1.00–1.12; women: model 2 OR 1.11; 95% CI, 1.05–1.18) were significantly associated with obesity in both sexes, and this association was more evident in women than in men.

### Interaction between number of teeth and food intake frequency

Table 3 shows the proportion of respondents in each food intake frequency category stratified by sex, number of teeth, and weight status. The proportion of respondents with daily intake of fruit/vegetables and fish/meat was higher in the group without weight loss than in the group with weight loss, regardless of number of teeth or gender. Among the subgroups without weight loss, the proportion of respondents with daily intake of fruit/vegetables was higher in those with normal weight than in those with underweight or obesity in men and in women with <20 teeth but not in women with ≥20 teeth. The proportion of respondents with daily intake of fish/meat was lower in those with obesity than in those with normal weight or underweight, regardless of number of teeth or gender.

**Table 3.** Proportion of respondents in each food intake frequency category stratified by sex, number of teeth, and weight status

		Men		Women	
	
		Number of teeth		Number of teeth	
	
		≥20	<20	≥20	<20
**Weight loss**		(%)			
Fruit/vegetable intake	Daily	76.7	69.3	83.9	77.2
	Not daily	23.3	30.7	16.1	22.8
Fish/meat intake	Daily	38.3	32.0	46.5	35.4
	Not daily	61.7	68.0	53.5	64.6
**No weight loss**					
Fruit/vegetable intake	Daily	80.4	72.8	88.9	82.6
	Not daily	19.6	27.2	11.1	17.4
Fish/meat intake	Daily	43.5	35.6	50.8	40.1
	Not daily	56.5	64.4	49.2	59.9
**No weight loss, underweight**					
Fruit/vegetable intake	Daily	80.1	72.0	91.2	81.5
	Not daily	19.9	28.0	8.8	18.5
Fish/meat intake	Daily	42.9	36.5	56.6	42.0
	Not daily	57.1	63.5	43.4	58.0
**No weight loss, normal weight**					
Fruit/vegetable intake	Daily	81.1	73.6	89.7	84.0
	Not daily	18.9	26.4	10.3	16.0
Fish/meat intake	Daily	44.1	35.9	51.6	41.2
	Not daily	55.9	64.1	48.4	58.8
**No weight loss, obesity**					
Fruit/vegetable intake	Daily	78.6	70.9	85.0	80.0
	Not daily	21.4	29.1	15.0	20.0
Fish/meat intake	Daily	41.5	34.3	45.9	37.4
	Not daily	58.5	65.7	54.1	62.6

Table 4 shows the ORs for weight loss, underweight, and obesity with food intake frequency stratified by number of teeth. No obvious interaction was observed in relation to weight loss and obesity in either sex or in relation to underweight in men. A significant interaction was observed in relation to underweight only in women. Infrequent fruit/vegetable intake showed a higher OR in women with fewer teeth (model 2 OR 1.17; 95% CI, 1.01–1.34) than in other groups (model 2 OR 0.82; 95% CI, 0.63–1.07), while infrequent fish/meat intake showed a lower OR in women with more teeth (model 2 OR 0.83; 95% CI, 0.71–0.96) than in other groups (model 2 OR 0.97; 95% CI, 0.87–1.09).

**Table 4.** Interaction between frequency of food intake and number of teeth in relation to weight loss, underweight, and obesity

		Number of teeth (Model 2^b^)						Interaction (*P*-value)		
	
		≥20			<20			Model 1^a^	Model 2^b^	Model 3^c^
	
		OR	95% CI		OR	95% CI				
**Weight loss, men**										
Fruit/vegetable intake	Not daily	1.14	1.001	1.31	1.12	1.03	1.21	0.61	0.78	0.83
Fish/meat intake	Not daily	1.21	1.09	1.36	1.15	1.06	1.24	0.47	0.42	0.38
**Weight loss, women**										
Fruit/vegetable intake	Not daily	1.47	1.25	1.73	1.30	1.19	1.42	0.26	0.22	0.20
Fish/meat intake	Not daily	1.14	1.02	1.28	1.22	1.13	1.32	0.57	0.30	0.27
**Underweight, men**										
Fruit/vegetable intake	Not daily	1.04	0.79	1.38	1.11	0.96	1.29	0.75	0.76	0.49
Fish/meat intake	Not daily	1.07	0.85	1.33	0.96	0.84	1.10	0.71	0.36	0.27
**Underweight, women**										
Fruit/vegetable intake	Not daily	0.82	0.63	1.07	1.17	1.01	1.34	0.01	0.01	0.08
Fish/meat intake	Not daily	0.83	0.71	0.96	0.97	0.87	1.09	0.04	0.046	0.19
**Obesity, men**										
Fruit/vegetable intake	Not daily	1.13	1.01	1.26	1.03	0.96	1.12	0.55	0.18	0.44
Fish/meat intake	Not daily	1.11	1.01	1.21	1.02	0.95	1.09	0.37	0.13	0.25
**Obesity, women**										
Fruit/vegetable intake	Not daily	1.33	1.14	1.54	1.20	1.10	1.31	0.03	0.16	0.24
Fish/meat intake	Not daily	1.09	0.99	1.21	1.10	1.02	1.18	0.17	0.99	0.96

CI, confidence interval; OR, odds ratio.

^a^Adjusted for age.

^b^Further adjusted for walking time, smoking, educational attainment, equivalent annual income, denture use and body mass index (only in the analysis for weight loss).

^c^Further adjusted for number of teeth, fruit/vegetable intake and fish/meat intake.

## DISCUSSION

Fewer teeth and infrequent food intake were associated with weight loss and obesity after adjusting for physical activity and other possible confounders in community-dwelling older men and women. In particular, these associations with weight loss were observed after adjusting for present BMI. Of note, associations between infrequent food intake and weight loss were observed among respondents with ≥20 teeth as well as among those with <20 teeth, suggesting that infrequent food intake may not just mediate the association between poor oral health and weight loss but be of intrinsic importance to it.

Investigating the relationship between poor oral health and risk of weight loss, Sullivan et al reported in a cross-sectional study that the number of general oral problems was associated with involuntary weight loss among inpatients of a geriatric rehabilitation unit.^4^ Weyant et al reported in a 2-year longitudinal cohort study that the OR for weight loss was 1.5 times higher among community-dwelling people aged ≥65 with periodontal disease than among those without it.^5^ Besides weight loss, several studies have reported associations of poor oral health with disability^8^^,^^9^ and mortality.^8^^,^^10^ Holm-Pedersen et al found the OR for disability was 3 times higher among older adults with tooth loss (1–9 teeth) than among those with more teeth (≥20) over 10 years of follow-up,^8^ and Aida et al^9^ found that the hazard ratio for functional disability was 1.2 times higher for subjects self-reporting <20 teeth than for those self-reporting ≥20 teeth over 4 years of follow-up. As for the association of poor oral health with mortality, hazard ratios were 1.3 times higher for edentulous subjects over 20 years of follow-up^8^ and 1.4 times higher for periodontal inflammation over 21 years of follow-up.^10^ Although the number of studies investigating the association of poor oral health with poor health outcomes in older people has been increasing, the biological mechanisms behind these relationships are not fully known.

Poor oral health has considerable influence on food choice, dietary intake,^11^^–^^14^^,^^18^ and nutritional status.^11^^,^^16^^,^^18^ People with fewer teeth seem to consume fewer fruit and vegetables, meat, beans, nuts, oil, and fish and shellfish,^11^^–^^14^^,^^18^ which can lead to poor nutritional status through lower intake of vitamins, carotenoids, dietary fiber, calcium, non-haem iron, and protein,^11^^,^^12^^,^^15^^,^^17^^,^^18^ as well as low blood concentration of vitamins, carotenoids, and albumin.^11^^,^^16^^,^^17^ Furthermore, tooth loss may be associated with higher intake of fat, cholesterol, alcohol, and added sugar, but not total energy.^13^^,^^15^^,^^18^ Even among dentists, for whom sufficient dental care is available, having fewer teeth was associated with poorer nutrition intake.^18^ These findings on the relationship between poor oral health and dietary intake suggest that dietary intake is just an intermediate factor in the association between poor oral health and weight loss; however, the present findings of associations between weight loss and intake of fruit/vegetables and fish/meat both among respondents with ≥20 teeth and among those with <20 teeth suggests that food intake is not merely a mediator but an independent determinant of weight loss.

The associations between having fewer teeth, food intake, and stable underweight were somewhat different from their associations with weight loss. This study revealed that having fewer teeth was associated with underweight in men but not in women. Previous studies have reported that poor oral health, such as having reduced clinical oral functions,^18^ self-perceived poor chewing abilities,^19^ or being edentate/not wearing dentures^20^ was associated with underweight status. However, dietary intake was not investigated in these studies. Infrequent intake of fruit/vegetables and fish/meat was associated with weight loss in both sexes in the present study, suggesting that older people experiencing recent weight loss may, in general, eat smaller amounts of many types of foods for various reasons. On the other hand, stable underweight status was marginally associated with infrequent fruit/vegetable intake only in men and, somewhat surprisingly, with frequent fish/meat intake in women. We suppose that this latter association might have been caused by evaluating the combined intake of fish and meat in one questionnaire item rather than analyzing their intake separately. The findings for fish/meat intake in the present study might, in fact, reflect a larger proportion of fish than meat intake, given that Japanese older people eat more fish than meat.^29^ A recent double-blinded, randomized, parallel intervention study^31^ found an appetite-suppressing effect of docosahexaenoic acid (DHA)-rich fish oil ingestion, which resulted in reduced energy intake, and it is possible that habitual frequent intake of fish is associated with a lower body weight, although the study reported a borderline association of frequent fish intake with decline in body weight. Further detailed study of the association between fish intake and underweight status is warranted.

Another notable finding of this study is the interaction effect observed between having fewer teeth and fruit/vegetable intake in relation to underweight in women; specifically, infrequent fruit/vegetable intake showed an OR of 1.2 for underweight in women with fewer (<20) teeth, but no significant association was observed in women with more teeth (≥20). This result suggests the importance of a preventive dietary approach for women with poor oral health. To prevent underweight in older people, especially those with poor oral health, a dietary approach that involves special cooking and processing of foods might be worthwhile. Further detailed study is also needed to clarify the association between tooth loss, food intake, and underweight.

Having fewer teeth and a lower food intake were also associated with obesity in this study. Several studies have reported an association between poor oral health and obesity.^20^^–^^25^ Hilgert et al demonstrated that edentulous persons wearing upper dentures and dentate persons with 1–8 teeth wearing one or no dentures showed ORs of 2.3 and 3.0, respectively, for obesity compared with dentate persons with >8 teeth among older people living independently in the community.^22^ De Marchi et al reported that community-dwelling older people with >8 teeth were 0.5 times less likely to have central obesity than edentulous person wearing two dentures.^25^ Tôrres et al found that edentate persons not wearing dentures had an OR for overweight and obesity of 2.9 compared to those with ≥20 teeth with a prosthesis.^21^ In addition, Ekback et al revealed that older adults with self-perceived good chewing capacity were less likely to be obese,^20^ and Ikebe et al reported that poor oral clinical function was associated with overweight.^19^ However, Ostberg et al observed that tooth loss (<20 teeth) was positively associated with obesity in younger adults aged 30–59 years, but no similar association was found in older adults aged 60–74 years.^23^ Our results support a positive association between having fewer teeth and obesity. However, many studies, including ours, involved cross-sectional analysis. It is possible that having fewer teeth increases the risk of obesity; however, it is also possible that comorbidity with obesity (eg, diabetes) can increase the risk of having fewer teeth. Jimenez reported that men with type 2 diabetes had hazard ratios of 1.3 for incidence of periodontitis and 1.1 for onset of tooth loss in a 20-year follow-up study.^32^ Moreover, they found a higher risk of periodontitis among people with lower intake of fruit and vegetables. Further longitudinal study is needed to clarify the association between having fewer teeth, dietary intake, and obesity.

In this study, no interaction effect was observed between having fewer teeth and fruit/vegetable intake in relation to obesity. Among the various foods available, the low-energy-density characteristic of fruit and vegetables is thought to be favorable for reducing body weight.^33^^,^^34^ In a large cross-sectional study conducted in the United States, the proportion of people who consumed >5 servings of fruit and vegetables per day was lower among those who were overweight and obese than among those of normal weight.^34^ A review of intervention studies in adults suggests that a low-energy-dense diet (increased fruit and vegetable content and decreased fat content) should effectively decrease body weight without a feeling of deprivation because fruit provides satiety.^33^ An abundant fruit and vegetable intake would therefore seem necessary to control obesity regardless of the number of remaining teeth.

This study has several strengths. First, it included a large number of community-dwelling older people from many areas across Japan, so the data analyzed should be reasonably representative of this population in Japan. Second, we evaluated both weight loss and stable underweight, because these represent different statuses and are important predictive factors for disability and mortality in older people.^1^^,^^2^ Third, oral health, food intake, and their interaction were considered while adjusting for potential confounders in the statistical models.

We also recognize some limitations of the study. First, causal relationships cannot be inferred due to the study’s cross-sectional design, so we could not identify whether having fewer teeth and infrequent food intake were the cause or result of weight loss and BMI status in this study. Second, although we considered a number of potential confounders, other factors, such as total energy intake and neighborhood environment,^35^ should be further considered. We could not consider total energy intake in this study because of a lack of data. If we could adjust for total energy intake in the statistical model, it could be helpful in understanding the observed associations (eg, the association between infrequent intake of fish/meat and obesity). Third, some measurement bias is possible because of the use of self-reported data for weight loss, height, body weight, and food intake frequency. In the JAGES project, we could not conduct a comprehensive dietary survey because it was a large-scale study of older subjects that included a considerable number of respondents aged >80 years, which made it difficult to administer a full-length, validated food frequency questionnaire or other dietary surveys, such as dietary records or dietary recall. Such dietary surveys will often be completed only by the healthier older population, so introducing these dietary surveys will result in more selection bias. A recent report by Cook et al^36^ demonstrated that answering a single questionnaire item on the consumption of fruit was a suitable replacement to answering a longer validated food frequency questionnaire when the objective was only to detect inadequate intake and assess population mean intake (ie, not to detect individual intake). We opted not to use a simple question to assess the amount of food consumed and instead assessed frequency as daily intake or not. However, we cannot deny this may still include some element of misclassification.

In conclusion, this study found that having fewer teeth was associated with both weight loss and obesity in both men and women and with underweight in men. Frequent food intake is a possible preventive factor against weight loss, regardless of the number of remaining teeth, and implementing special cooking and food processing of foods may be an effective countermeasure for preventing underweight status in women with poor oral health. The associations observed in this cross-sectional study should be verified by longitudinal and interventional studies.

## ONLINE ONLY MATERIAL

Abstract in Japanese.
