# Supplementary material for: Poor Oral Health and Diet in Relation to Weight Loss, Stable Underweight, and Obesity in Community-Dwelling Older Adults: A Cross-Sectional Study From the JAGES 2010 Project
Source: J Epidemiol. 2016 Jun 5;26(6):322–9. doi: 10.2188/jea.JE20150144 (PMC4884901; doi:10.2188/jea.JE20150144)
Supplement: Abstract in Japanese. [file je-26-322-s001.pdf]

地域在住高齢者における歯の不健康･食事と体重減少、安定した痩せ、肥満との関連:JAGES 2010 プロジェクト横断研究

中村　美詠子（浜松医科大学健康社会医学講座）
尾島　俊之（浜松医科大学健康社会医学講座）
中出　美代（東海学園大学健康栄養学部管理栄養学科）
大塚　理加（国立研究開発法人国立長寿医療研究センター在宅連携医療部）
山本 龍生（神奈川歯科大学大学院歯学研究科社会歯科学講座）
鈴木　佳代（愛知学院大学総合政策学部）
近藤　克則（千葉大学予防医学センター）

背景：意図しない体重減少や痩せは高齢者の死亡や障害のリスクを増加させる。しかし歯の不健康・食事とボディマスインデックス（BMI）との関連や交互作用は解明されていない。
方法：日本老年学的評価研究において日本の 31 市町から無作為に抽出された 65 歳以上の回答者 96,794 人のデータを分析した。体重減少は調査時点に先立つ半年間における 2-3kg 以上の減少と定義した。BMI は体重減少がない者について評価した。体重減少、痩せ、肥満を従属変数、少ない歯数（20 本未満)、食品の低頻度摂取を独立変数として、交絡因子を調整した多重ロジスティック回帰分析を実施した。
結果：体重減少は少ない歯数（男性オッズ比（OR）1.3、95%信頼区間（CI）1.2-1.3、女性 OR 1.2, 95% CI 1.1-1.3)、果物・野菜の低頻度摂取(男性 OR 1.1、 95% CI 1.1-1.2、 女性 OR 1.4、95% CI1.3-1.5)、魚・肉の低頻度摂取(男女 OR 1.2, 95% CI 1.1-1.3)と関連していた。少ない歯数と食品摂取の間の交互作用は見られなかった。肥満も同様の要因と関連していた。すなわち少ない歯数(男性 OR 1.2、女性 OR 1.3)、果物・野菜の低頻度摂取(男性 OR 1.1、女性 OR 1.2)、魚・肉の低頻度摂取性（男女 OR 1.1)であった。歯数が少ない者はそうでない者に比べ、果物・野菜の低頻度摂取は女性の痩せに対して高いオッズ比を示した。
結論：少ない歯数と食品の低頻度摂取は体重減少と肥満の両方に関連し、女性の痩せとの関連では有意な交互作用が観察された。

痩せ、高齢者、少ない歯数、食品、果物と野菜
